# Comparison of anastomotic stricture rates between 23- and 25-mm powered circular staplers in cervical esophagogastric anastomosis: a propensity-matched study

**DOI:** 10.1007/s10388-025-01171-2

**Published:** 2025-12-22

**Authors:** Akira Saito, Koji Otsuka, Satoru Goto, Tomotake Ariyoshi, Takeshi Yamashita, Kentaro Motegi, Masahiro Komoto, Yutaka Kishimoto, Masahiko Murakami, Takeshi Aoki

**Affiliations:** 1 Division of General and Gastroenterological Surgery, Department of Surgery, Showa University School of Medicine, 1-5-8, Hatanodai, Shinagawa, Tokyo, 142-8666 Japan; 2 Digestive Diseases Center, Showa University Koto Toyosu Hospital, Tokyo, Japan; 3 Esophageal Cancer Center, Showa University Hospital, Tokyo, Japan

**Keywords:** Esophagectomy, Powered circular stapler, Anastomotic stricture, Propensity score matching, Anastomotic leakage

## Abstract

**Background:**

Circular stapler (CS) anastomosis is widely used in McKeown esophagectomy; however, anastomotic stenosis remains a significant concern. This study aimed to compare stenosis rates between newly introduced 23- and 25-mm powered CSs in cervical esophagogastric anastomosis.

**Methods:**

From May 2022 to February 2024, 126 patients who underwent thoracoscopic McKeown esophagectomy with retrosternal gastric conduit reconstruction were retrospectively analyzed. They were categorized into the 23-mm (*n* = 52) and 25-mm (*n* = 74) CS groups. The primary endpoint was the comparison of anastomotic stricture rates. Propensity score matching was performed to adjust for potential confounders.

**Results:**

After propensity score matching, 39 pairs were selected. The incidence of anastomotic stricture was 18.0% (7 cases) and 12.8% (5 cases) in the 23- and 25-mm CS groups, respectively (*p* = not significant [NS]). The incidence of anastomotic leakage and the leakage location revealed no significant differences between the two groups. A two one-sided test for equivalence was performed to compare the risk difference in stricture rates between the groups, resulting in a risk difference of 5.1% with a 90% confidence interval of − 0.087 to 0.191 and a p value of 0.041, suggesting potential equivalence.

**Conclusion:**

In this study, 23- and 25-mm powered CSs achieved comparable anastomotic stricture rates in cervical esophagogastric anastomosis during McKeown esophagectomy. The newly introduced 23-mm powered CS can be a useful option, considering its easier anvil insertion into the residual esophagus when insertion of the 25-mm CS anvil is technically challenging.

## Introduction

McKeown esophagectomy is a standard surgical procedure for thoracic esophageal cancer, with various options available for cervical esophagogastric conduit anastomosis. These options include circular stapler (CS) anastomosis, side-to-side linear staple anastomosis, the Collard method, triangulating stapling, and hand-sewn anastomosis [1–4]. Among these, CS anastomosis offers significant advantages: it is technically simple and achieves consistent anastomotic leakage rates and is suitable for young esophageal surgeons regardless of their experience [5, 6]. However, anastomotic stricture, which occurs more frequently with CS anastomosis than with other methods, has been reported to occur in 3.8–53% of cases [7–11]. This complication cannot be overlooked, as it significantly affects patients’ postoperative quality of life, often necessitating repeated endoscopic balloon dilations and potentially causing aspiration pneumonia due to dysphagia.

Previous studies have investigated the relationship between anastomotic stricture and CS diameter, yielding conflicting results; some suggest that larger CS diameters are associated with lower stricture rates, whereas others report little correlation [11, 12]. Recently, the introduction of powered CS, which reportedly provides a more stable anastomosis than manual CS, along with the availability of 23-mm-diameter options, has expanded both stapler specifications and diameter choices [13].

In our clinical practice, before the introduction of the 23-mm powered CS, the 21-mm CS was the only alternative when inserting a 25-mm anvil was challenging. However, based on our experience with frequent postoperative anastomotic strictures using the 21-mm CS, we prioritized the use of the 25-mm CS whenever possible, even in cases with a narrow residual esophagus, using dilation techniques. The availability of the 23-mm CS has now expanded our options, prompting us to evaluate its utility, particularly given its smaller diameter, which facilitates easier anvil insertion.

Therefore, this study aimed to compare the postoperative anastomotic stricture rates between 23- and 25-mm powered CSs in cervical esophagogastric anastomosis during esophageal cancer surgery, using identical stapler specifications, to evaluate the utility of the newly introduced 23-mm powered CS.

## Methods

### Study design and patients

This single-center, retrospective cohort study was conducted at the Showa University Hospital, Tokyo, Japan. From May 2022 to February 2024, we reviewed 188 consecutive patients who underwent thoracoscopic McKeown esophagectomy (including robot-assisted surgery). We excluded patients with cervical esophageal cancer (*n* = 15), those using CSs other than the Echelon Circular Powered Stapler (Echelon Circular™ Powered Stapler, Ethicon Inc., Cincinnati, OH, USA) (*n* = 17), those who underwent colonic reconstruction (*n* = 8), those who underwent posterior mediastinal reconstruction (*n* = 9), those who underwent hand-sewn anastomosis (*n* = 7), those with esophageal achalasia (*n* = 4), and those who were lost to follow-up (*n* = 2). Finally, 126 patients who underwent cervical esophagogastric anastomosis with retrosternal reconstruction were included in the analysis.

CSs are available in manual and powered varieties with various diameters. A previous study reported that powered staplers result in fewer anastomotic leaks than manual staplers of the same diameter [13]. Additionally, anastomotic leakages have been reported to increase the anastomotic stricture rate [14]. Manual and powered staplers also differ in terms of staple formation and tissue compression rates. Therefore, to accurately compare stricture rates between the 23- and 25-mm powered CSs, it is necessary to limit the comparison to powered CSs only. Thus, in this study, only cases wherein a 23- or 25-mm powered CS was used were included.

We collected patient characteristics, including age; sex; body mass index (BMI); presence of cardiac disease, chronic obstructive pulmonary disease (COPD), or diabetes mellitus (DM); clinical stage (TNM Classification, 8th edition); tumor location; preoperative therapy; esophageal diameter (at the level of the lower pole of the thyroid on preoperative computed tomography [CT]); surgical procedure; extent of lymphadenectomy; and preoperative histological type. Moreover, perioperative data included operation time; blood loss; anastomotic leakage (≥Clavien–Dindo Classification [CD] IIIa); leakage location (esophagogastric anastomosis or blind end of the gastric conduit); anastomotic stricture (≥ CD IIIa); postoperative hospital stay; and timing of stricture dilation [15, 16].

### CS selection

The Echelon Circular Powered Stapler with a diameter of 23 mm or 25 mm was used. The selection of the CS size depends on the diameter of the residual esophagus, making it difficult to standardize the size selection preoperatively. Therefore, in this study, the diameter of the residual esophagus was primarily measured at the time of anvil insertion, and the 25-mm CS was used when the insertion was easy. The criterion for “ease of insertion” was left to the surgeon’s discretion.

### Definition of the anastomotic stricture

Anastomotic stricture was defined as cases requiring endoscopic balloon dilation due to difficulty in passing a standard endoscope (9.9-mm diameter) during symptomatic episodes or postoperative surveillance (at 1 year).

### Surgical technique

**Fig. 1:**
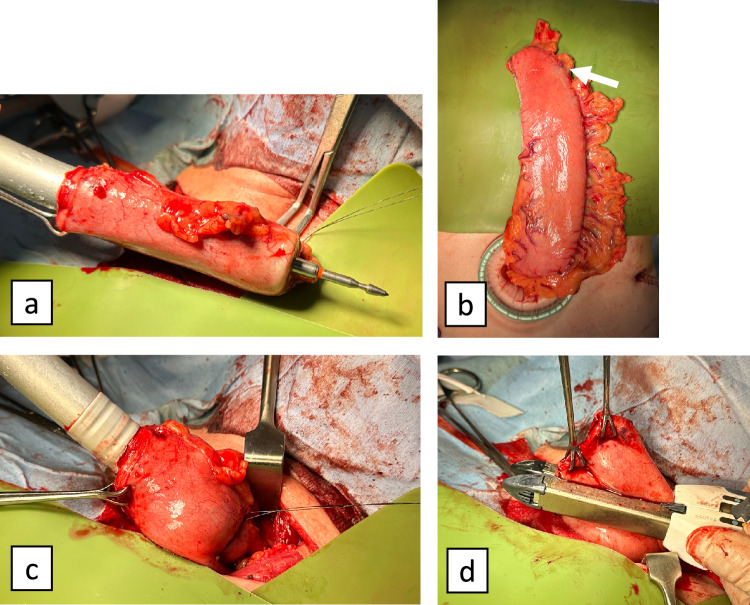
Anastomotic technique. **a** The circular stapler rod tip was positioned as close as possible to the greater curvature on the anal side. **b** The white arrow indicates the anastomosis site. **c** The entry hole of the gastric conduit was completely relaxed to achieve a tension-free state before connecting the rod and anvil. **d** The entry hole was closed using a linear stapler and reinforced by burying the staple line with 3-0 absorbable sutures

All patients underwent thoracoscopic (including robot-assisted) McKeown esophagectomy [17, 18]. The gastric conduit was created using the hand-assisted laparoscopic surgery technique with three ports and a 7-cm transverse incision in the upper abdomen while preserving the right gastroepiploic and right gastric arteries. A subtotal gastric conduit with a diameter of 5 cm was created using linear staplers. The conduit was then pulled up through the retrosternal route to the neck, with the CS rod tip positioned as close to the greater curvature anal side as possible, considering the gastric conduit blood flow. In all cases, to facilitate anvil insertion, we inserted intestinal forceps (using forceps with a wide tip to distribute pressure within the esophageal lumen during dilation) approximately 2 cm into the residual esophagus and gently dilated the lumen prior to insertion. During anastomosis, the entry hole of the gastric conduit was completely relaxed to achieve a tension-free state before the rod and anvil were connected in a side-to-end configuration. After the anastomosis, the entry hole was closed using a linear stapler, and the stump was reinforced by burying it with 3-0 absorbable sutures (Fig. 1). Although eight surgeons performed the operations, the procedure was standardized and manualized, and at least one of the five qualified surgeons (Japanese endoscopic surgical skill qualification system) participated in all operations as the responsible surgeon [19].

### Evaluation criteria

Patient characteristics and perioperative data were compared between the 23- and 25-mm powered CS groups. In this retrospective study, the primary endpoint was to compare the stricture rates between the two groups, whereas the secondary endpoint was to assess the risk difference in stricture rates between the groups.

### Propensity score matching (PSM) and statistical analysis

To minimize background bias, 1:1 PSM was performed for the following factors potentially associated with anastomotic stricture: age, sex, BMI, cardiac disease, COPD, DM, cStage, tumor location, preoperative therapy, extent of lymphadenectomy, and preoperative histological type. The caliper width was set at 0.2 of the standard deviation of the propensity score logit. Furthermore, to confirm whether similar results would be obtained, inverse probability weighting (IPW) analysis was also performed.

Continuous variables are expressed as medians and interquartile ranges. Nonparametric variables were compared using the Mann–Whitney U test. Categorical data were compared using Fisher’s exact test. Based on previous reports indicating anastomotic stricture rates of 3.8–53%, and considering this study as a pilot, we set a clinically acceptable margin of ±20% [10, 11]. A two one-sided test (TOST) for equivalence was performed to assess the risk difference in stricture rates between the groups using 90% confidence intervals (CIs). A *p* value of < 0.05 was considered statistically significant. Statistical analysis was performed using JMP Pro 17 (SAS Institute Inc., Cary, NC, USA) and EZR (Jichi Medical University, Tochigi, Japan), which is a graphical user interface for R (The R Foundation for Statistical Computing, Vienna, Austria) [20].

## Results

In total, 126 patients were included in this study: 52 patients in the 23-mm CS group and 74 in the 25-mm CS group. The minimum follow-up period for this study was 9 months, and there were no cases of residual esophageal injury due to anvil insertion. Regarding baseline characteristics, the 23-mm CS group had a significantly higher prevalence of DM than the 25-mm CS group (11 [21%] vs. 6 [8%]; *p* = 0.031). After PSM, 39 patients were selected from each group. Only esophageal diameter was different between the groups (16.9 mm vs. 18.6 mm; standardized mean difference [SMD] = 0.401; *p* = 0.041), and other variables were well-balanced. Similar results were observed with IPW analysis (Table 1).

**Table 1:** Patient characteristics

Variable	All patients				Propensity-matched patients				Inverse probability weighting		
	23-mm CS group	25-mm CS group	*P* value	SMD	23-mm CS group	25-mm CS group	*P* value	SMD	23-mm CS group	25-mm CS group	SMD
	*n* = 52 (%)	*n* = 74 (%)			*n* = 39 (%)	*n* = 39 (%)			*n* = 125 (%)	*n* = 123 (%)	
Age, median [IQR]	68.5 [62–85]	68 [57–74]	0.261	0.182	68 [62–76]	71 [65–77]	0.357	0.229	68.5 [62–75]	68 [57–74]	0.026
Sex			0.056	0.343			0.575	0.127			0.025
Male	37 (71)	63 (85)			30 (77)	32 (82)			100 (80)	100 (81)	
Female	15 (29)	11 (15)			9 (23)	7 (18)			25 (20)	23 (19)	
BMI, median [IQR]	20.9 [19.1–22.9]	21.8 [19.5–24.0]	0.120	0.285	21 [19.4–23.3]	21.1 [18.8–23.3]	0.621	0.135	20.9 [19.1–22.9]	21.8 [19.5–24.0]	0.121
Comorbidity											
Cardiac disease	19 (37)	27 (36)	0.995	0.001	14 (36)	15 (38)	0.815	0.053	49 (39)	47 (38)	0.023
COPD	5 (10)	11 (15)	0.384	0.161	4 (10)	6 (15)	0.498	0.154	14 (12)	15 (12)	0.027
Diabetes mellitus	11 (21)	6 (8)	0.031	0.376	7 (18)	6 (15)	0.761	0.069	18 (15)	17 (14)	0.021
cStage			0.886	0.026			0.591	0.122			0.023
I	10 (19)	15 (20)			8 (21)	10 (26)			29 (23)	27 (22)	
II–IV	42 (81)	59 (80)			31 (79)	29 (74)			96 (77)	96 (78)	
Tumor location			0.347	0.266			1.00	<0.001			0.077
Upper	5 (10)	10 (14)			5 (13)	5 (13)			13 (11)	16 (13)	
Middle	30 (58)	33 (45)			20 (51)	20 (51)			67 (54)	62 (50)	
Lower	17 (32)	31 (42)			14 (36)	14 (36)			45 (36)	46 (37)	
Histological type			0.646	0.163			0.310	0.247			0.079
Squamous cell carcinoma	46 (88)	68 (92)			35 (90)	33 (85)			111 (89)	112 (91)	
Adenocarcinoma	4 (8)	5 (7)			4 (10)	5 (13)			11 (9)	9 (7)	
Others	2 (4)	1 (1)			0 (0)	1 (3)			3(2)	2(2)	
Preoperative therapy			0.263	0.294			0.839	0.135			0.155
None	6 (12)	14 (19)			6 (15)	8 (21)			17 (13)	20 (16)	
Neoadjuvant chemotherapy	42 (81)	56 (78)			31 (79)	29 (74)			98 (79)	98 (80)	
Chemoradiation	4 (8)	2 (3)			2 (5)	2 (5)			10 (8)	6 (4)	
Size of esophagus, mm, median [IQR]	16.6 [14.7–19.2]	18.1 [15.3–20.1]	0.084	0.244	16.9 [14.9–19.3]	18.6 [15.4–21.3]	0.041	0.401	16.6 [14.7–19.2]	18.1 [15.3–20.1]	0.591
Surgical procedure			0.278	0.196			0.615	0.114			0.006
MIE	34 (65)	55 (74)			27 (69)	29 (74)			92 (74)	91 (74)	
RAMIE	18 (35)	19 (26)			12 (31)	10 (26)			33 (26)	31 (26)	
Field of lymphadenectomy			0.101	0.142			1.00	<0.001			0.124
2FL	40 (77)	47 (64)			30 (77)	30 (77)			95 (76)	87 (71)	
3FL	12 (23)	27 (36)			9 (23)	9 (23)			30 (24)	36 (29)	

*IQR* Interquartile range, *BMI* Body mass index, *COPD* Chronic obstructive pulmonary disease, *MIE* Thoracoscopic McKeown esophagectomy, *RAMIE* Robot-assisted thoracoscopic McKeown esophagectomy, *FL* Field of lymphadenectomy

Surgical outcomes, including operation time and blood loss, showed no significant differences between the groups across the entire cohort, propensity-matched cohort, and IPW analysis. In the analysis of all patients, the incidence of anastomotic stricture was 17.3% (9 cases) in the 23-mm CS group and 14.9% (11 cases) in the 25-mm CS group (*p* = not significant [NS]). In the propensity-matched cohort, the incidence of anastomotic stricture was 18.0% (7 cases) and 12.8% (5 cases) in the 23- and 25-mm CS groups, respectively (*p* = NS). In IPW analysis, the incidence of anastomotic stricture was 21% (26 cases) and 13% (16 cases) in the 23- and 25-mm CS groups, respectively (*p* = NS). The incidence of anastomotic leakage, the leakage location, and the timing of stricture dilation showed no significant differences between the two groups in all patients, propensity-matched patients, and IPW analysis; however, there was a tendency for earlier timing of stricture dilation in the 23mm CS group (Table 2).

**Table 2:** Surgical outcomes and post operative complications

Variable	All patients			Propensity-matched patients			Inverse probability weighting		
	23-mm CS group	25-mm CS group	*P* value	23-mm CS group	25-mm CS group	*P* value	23-mm CS group	25-mm CS group	*P* value
	*n* = 52 (%)	*n* = 74 (%)		*n* = 39 (%)	*n* = 39 (%)		*n* = 125 (%)	*n* = 123 (%)	
Operation time, min, median [IQR]	483 [388–544]	497 [418–657]	0.149	483 [388–544]	473 [401–545]	0.525	483 [388–544]	497 [418–600]	0.201
Blood loss, ml, median [IQR]	121 [77–232]	180 [98–319]	0.053	118 [86–242]	151 [72–226]	0.334	121 [77–232]	180 [98–319]	0.441
Anastomotic leakage	3 (5.8)	3 (4.1)	0.656	3 (7.7)	1 (2.6)	0.305	7 (5)	4 (3)	0.514
Leakage location			0.193			0.305			0.514
Esophagogastric anastomosis	3 (5.8)	1 (1.4)		3 (7.7)	1 (2.6)		7 (5)	1 (1)	
Blind end of gastric conduit	0 (0)	2 (2.7)		0 (0)	0 (0)		0 (0)	2 (2)	
Anastomotic stricture	9 (17.3)	11 (14.9)	0.712	7 (18.0)	5 (12.8)	0.530	26 (21)	16 (13)	0.289
Postoperative hospital stay, day, Median [IQR]	15.5 [12–23.5]	14 [11–22]	0.111	15 [12–24]	19 [11–24]	0.453	16 [12–24]	14 [11–22]	0.823
Timing of stricture dilation, day, median [IQR]	45 [32–96]	113 [38–141]	0.556	65 [34–114]	113 [97–133]	0.074	46 [34–96]	113 [38–141	0.129

*IQR* Interquartile range

A TOST for equivalence was performed to compare the risk difference in stricture rates between the groups, yielding a risk difference of 5.1% with a 90% CI of − 0.087 to 0.191 and a *p* value of 0.041, suggesting potential equivalence between the two groups (Table 3).

**Table 3:** TOST for equivalence comparing the risk difference in stricture rates between the 23- and 25-mm CS groups

Risk difference	Lower 90% CI	Upper 90% CI	*P* value
0.051	− 0.087	0.191	0.041

*TOST* Two one-sided test, *CI* Confidence interval

## Discussion

In this retrospective study using PSM, the anastomotic stricture rates following cervical esophagogastric anastomosis were 18.0% in the 23-mm CS group and 12.8% in the 25-mm CS group, with no significant difference between the groups. In IPW analysis, no significant differences were observed. A TOST for equivalence comparing the risk difference in stricture rates between the groups yielded a risk difference of 5.1%, with a 90% CI of − 0.087 to 0.191 and a p value of 0.041, suggesting potential equivalence. However, although no statistically significant difference in anastomotic stricture rates was observed between the different CS sizes used, a trend toward increased stricture rates and earlier timing of stricture dilation was observed in the 23-mm CS group. While the extent to which the difference in early versus late timing of dilation procedures affects patient outcomes remains unclear, the trend toward increased stricture rates cannot be ignored. Therefore, the use of 23-mm CS is not recommended for all cases, and the 23-mm CS may be a useful alternative when insertion of the 25-mm CS anvil is technically challenging.

Previous studies have identified several risk factors for CS anastomotic stricture, including older age, high BMI, COPD, DM, cardiovascular disease, upper thoracic tumor location, and neoadjuvant therapy [9, 11, 21–24]. To evaluate the effects of CS diameter exclusively, we performed PSM to minimize selection bias by standardizing these background factors. Anastomotic leakage is a major risk factor for stricture, as it leads to tissue necrosis and structural collapse at the esophagogastric anastomosis site. During healing, fibroblasts in the granulation phase produce collagen and fibronectin to reconstitute the tissue, followed by re-epithelialization. Contraction occurs simultaneously, with myofibroblasts recruited around the wound exerting tension that reduces the anastomotic diameter [10, 25]. In our study, overall leakage rates did not differ significantly between the groups, although they were slightly higher in the 23-mm CS group, which could have been disadvantageous. Therefore, the influence of anastomotic leakage on stricture formation was minimized, supporting the validity of our comparative results.

Regarding surgical factors, while some reports identify smaller CS as a risk factor for anastomotic stricture, other reports suggest that CS size is not related. Hosoi et al. reported stricture rates of 53% and 23% for 25 mm and 28 or 29 mm, respectively, indicating smaller diameters as a risk factor [11]. However, in clinical practice, the insertion of a larger anvil is often technically challenging, compelling surgeons to choose smaller diameters despite higher stricture risks. On the other hand, Wang J et al. compared anastomotic stricture rates between 21-mm and 25-mm CSs using PSM and reported stricture rates of 12.3% and 7%, respectively, with no statistically significant difference [12]. This finding indicates the absence of significant difference and does not prove equivalence or noninferiority of 21-mm CS compared with 25-mm CS. The trend toward higher stricture rates with 21-mm CS, while recommending the use of 23-mm CS when 25-mm CS insertion is difficult, supports our conclusion. The report also mentions the risk of residual esophageal injury associated with larger anvil insertion, which can be considered a common concern among esophageal surgeons using CS for anastomosis. Theoretically, CS with larger anastomotic diameter should be less prone to stricture compared with CS with smaller anastomotic diameter. Regarding the stapler comparison, the inner diameters of the staples (knife inner diameter) for 23-mm CS and 25-mm CS are 14.6 mm and 16.5 mm, respectively, with a difference of 1.9 mm. In this study, anastomotic stricture was defined as "cases requiring balloon dilation when a standard endoscope with a 9.9-mm diameter could not pass through." By this definition, the 1.9-mm difference in inner diameter does not appear to be a major contributing factor to anastomotic stricture. However, this applies when appropriate anvil size is selected to avoid vascular compromise or excessive mucosal tension during anvil insertion, and stable anastomotic technique is performed. Regarding other surgical risk factors for anastomotic stricture, Shiraishi et al. identified gastric conduit tension as a risk factor, thereby reducing the stricture rate from 29.1% to 3.8% using a nontensioning method [10]. We standardized the tension-free anastomosis by completely relaxing the gastric conduit entry hole during anastomosis in all cases, thereby eliminating any anastomotic technique bias.

The esophagus typically measures 20–30 mm in diameter; however, considerable individual variation exists, and proximal esophageal dilation is often observed in cases with cancerous strictures. This variability in esophageal diameter complicates the optimal selection of CS size. In this study, we measured the preoperative esophageal diameter via CT and found that the 25-mm group had larger esophageal diameters. This outcome was expected, given the use of the largest available CS; however, no significant difference in stricture rates was observed. Larger CS sizes do not necessarily reduce anastomotic strictures, likely because various conditions of both the residual esophagus and gastric conduit affect stricture development. Although we previously attempted to use a larger CS, this study demonstrated the utility of a 23-mm CS, which enables easier insertion with less operator stress, enabling more appropriate CS size selection without excessive manipulation.

This study has several limitations. First, its retrospective nature may have introduced bias, although we attempted to minimize this using PSM. Second, it was a single-center study with a relatively small sample size (126 patients), partly because we excluded pre-23-mm CS introduction cases to avoid time-related bias. However, limiting the study to concurrent cases at a single institution may have reduced unmeasured bias by ensuring consistent surgical techniques and postoperative management. Third, because the 25-mm CS was selected when anvil insertion was easy, the utility of 23-mm CSs in all cases, particularly those with larger residual esophageal diameters, remains unclear. Further investigation of the optimal CS size, regardless of the residual esophageal diameter, is warranted. Fourth, blood flow evaluation of the gastric conduit at the anastomotic site was not performed. Although the usefulness of ICG fluorescence imaging for evaluating gastric conduit blood flow has been reported, standardized techniques are yet to be established, and we were unable to evaluate this in the present study [26–28]. However, it should be noted that in this study, subtotal gastric conduits were created in the presence of qualified surgeons in all cases while preserving the right gastric artery and right gastroepiploic artery, and similar surgical techniques were employed. Nevertheless, as a pilot study, this research successfully demonstrated the utility of the newly introduced 23-mm powered CS under specific conditions. Further prospective studies with larger sample sizes are necessary to provide more robust evidence.

## Conclusion

This retrospective study suggests that 23- and 25-mm powered CSs achieve comparable anastomotic stricture rates in cervical esophagogastric anastomosis during McKeown esophagectomy. The newly introduced 23-mm powered CS can be a useful option, considering its easier anvil insertion into the residual esophagus when insertion of the 25-mm CS anvil is technically challenging.
